# Estimating Neuronal Information: Logarithmic Binning of Neuronal Inter-Spike Intervals

**DOI:** 10.3390/e13020485

**Published:** 2011-02-01

**Authors:** Alan D. Dorval

**Affiliations:** Department of Bioengineering and the Brain Institute, University of Utah, Salt Lake City, UT 84108, USA

**Keywords:** neuron, neural information, inter-spike interval, logarithmic probability density, spike pattern entropy

## Abstract

Neurons communicate via the relative timing of all-or-none biophysical signals called spikes. For statistical analysis, the time between spikes can be accumulated into inter-spike interval histograms. Information theoretic measures have been estimated from these histograms to assess how information varies across organisms, neural systems, and disease conditions. Because neurons are computational units that, to the extent they process time, work not by discrete clock ticks but by the exponential decays of numerous intrinsic variables, we propose that neuronal information measures scale more naturally with the logarithm of time. For the types of inter-spike interval distributions that best describe neuronal activity, the logarithm of time enables fewer bins to capture the salient features of the distributions. Thus, discretizing the logarithm of inter-spike intervals, as compared to the inter-spike intervals themselves, yields histograms that enable more accurate entropy and information estimates for fewer bins and less data. Additionally, as distribution parameters vary, the entropy and information calculated from the logarithm of the inter-spike intervals are substantially better behaved, e.g., entropy is independent of mean rate, and information is equally affected by rate gains and divisions. Thus, when compiling neuronal data for subsequent information analysis, the logarithm of the inter-spike intervals is preferred, over the untransformed inter-spike intervals, because it yields better information estimates and is likely more similar to the construction used by nature herself.

## 1. Introduction

Neurons transmit signals via high amplitude, brief duration electrical pulses called spikes. While slow and anatomically local chemical signals play a minor role in neuronal communication, most of the information a neuron shares with its near and distant neighbors is conveyed by the timing of these spikes. Information theoretic analysis was first applied to these spikes, to estimate information transmission between neurons [1], only a few years after its formalization by Shannon [2]. Subsequent work has demonstrated the need for information theoretic approaches within neuroscience, for example, by proving that specific temporal patterning conveys more information than the average spike rate in motor neurons responsible for movement coordination [3]. However, information analysis remains a niche field within neuroscience, used primarily to quantify neuronal behaviors in response to time locked inputs that can be repeated hundreds of times. For example, the ability to rapidly repeat successive trials of visual scenes has made the visual system particularly amenable to information analyses, revealing that sensory neurons use different temporal scales to encode different classes of information [4,5], and that natural sensory events coupled with intrinsic dynamics enable neurons to share more information than well controlled laboratory experiments might suggest [6,7]. A more complete utilization of information theoretic tools would use simultaneously recorded neuronal inputs and outputs in natural conditions. Eventually, information theory will markedly impact both basic neuroscience and engineering neural prostheses. First however, tools and intuition must be developed to interpret neuronal signals, understand neuronal entropy, and assess neuronal information processing.

Unlike manufactured computational systems, neurons do not operate on a linear clock. Synaptic inputs modify internal states of a neuron, changing the membrane potential and voltage sensitive conductance gates. These changes do not last for long, with the internal states rapidly returning to their rest value through first, second or higher order kinetics. Indeed, the conductance gates recover according to a number of exponential mechanisms, the combinations of which approximate power law behavior [8]. When the relative timing of events obey power law relationships, a histogram of the logarithm of the time between events provides an intuitive appreciation for the time delays between events [9,10]. In recent work, we proposed that the logarithm of time is not only more intuitive, but is the natural metric for neuronal operations [11]: that neurons operate on a logarithmic clock suited to measure the scale of inter-spike intervals (ISIs, *i.e.*, the time between two spikes) better than their absolute durations. If true, then relative ISI changes should carry more information than absolute changes. Indeed, the difference between 1 and 2 ms ISIs likely conveys substantially more information than the difference between 101 and 102 ms ISIs.

Although linearly binned ISI histograms are more widespread, logarithmically binned ISI histograms have been used to classify neuronal bursting behavior [12] and to quantify changes in neuronal entropy between diseased and treated conditions [13]. In previous work, we explored the formal differences between these two histograms by taking the derivative of the cumulative distribution function with respect to either time or the logarithm of time, and then binning the respective probability density functions (PDFs). As a function of dataset size, entropies estimated from the logarithmic PDFs were less biased and less variable than from the more traditionally used linear PDFs. The PDF construction aside, many others have explored the amount of data needed to estimate entropy and information according to different techniques [14,15]. A generic finding is that, the more bins one uses to discretize the PDF, the more data is needed to appropriately estimate the probability associated with each bin. Thus, when discretizing ISIs for information analysis, one should use the least number of bins that enable correct subsequent information estimates.

In this work we will explore the bins needed to robustly estimate entropy and information from ideal ISI distributions. Too few bins and the salient features of the distribution are obscured; too many bins and it becomes impractical to collect enough data to fill them all adequately. We show that for the types of distributions that best describe neuronal activity, ISIs binned in logarithmic time enable fewer bins to capture the distribution salience. We find that for information analysis, the logarithmic PDFs are preferred over the linear PDFs because sufficiently accurate entropy and information can be found for fewer bins, and entropy and information are better behaved as a function of distribution parameters.

## 2. Results and Discussion

This section begins with a review of the differences between the linear and logarithmic probability density functions (PDFs), generated by differentiating a cumulative distribution function with respect to either its abscissa variable (e.g., *t*) or the logarithm of its abscissa variable (e.g., *log t*), respectively. Four distributions that model inter-spike intervals (ISIs) are used to examine the limits of entropy and information estimation from binned PDFs of both types. The section concludes with examinations of how bin count, distribution mean, and distribution variability affect entropy and information. Computer analyses were performed in the Octave numerical computing language.

### 2.1. Separate but Equal Probability Density Functions

When calculating entropy from data collected into K bins, estimate bias and variability are minimized when the probability associated with each bin approximates ~1/K. Binned PDFs with high and low probability events require more data to yield reliable entropy estimates than relatively uniform density functions [11]. Additionally, the more uniform the underlying PDF, the fewer the bins needed to construct a discretized version that captures the salient features of the true density. For these two related reasons—estimation accuracy and bin reduction—we seek relatively flat probability densities.

To begin, we assume that neuronal ISIs are independent. Although usually false, the entropy and information estimation techniques that we will use on single ISIs work equally well for ISI pairs, triplets, etc., with the caveat that higher order estimates require more data [16]. Others have shown how the results of a few low-order estimates (e.g., singles, pairs and triplets) can be extrapolated to approximate the true entropy and information [14]. In this manuscript, ISIs are strictly independent.

The time between successive spikes is drawn from a distribution function, *F(t) = P(ISI* ≤ *t)*. Introducing a variable *τ* ≡ *log(t)*, for strictly positive *t* we can substitute *t = 10^τ^* into the distribution function and take the derivative with respect to either *t* or *τ* to yield two different PDFs:

(1) $$\frac{d}{dt}(F(t))=\frac{dF}{dt}$$

(2) $$\frac{d}{d\tau }(F(10^{\tau }))=\frac{d}{d\tau }(10^{\tau })\frac{d}{dt}(F(t))=ln(10)10^{\tau }\frac{dF}{dt}=ln(10)\left(t\frac{dF}{dt}\right)$$

The logarithmic PDF is the linear PDF scaled by *t* and a constant, *ln(10)*. This temporal scaling results in density increases for large *t* and decreases for small *t*. For distributions with substantial negative skewness, scaling by *t* would exaggerate the regions of high probability and diminish the regions of low probability. Entropy and information calculations would then require finer binning and more data, or include more bias and variability. For distributions with minimal skewness, scaling by *t* would generate high and low probability regions, creating those same entropy—and information-estimation difficulties. Thus, logarithmic PDFs should be avoided when estimating information measures from negatively or negligibly skewed distributions. However, the linear PDFs of positively skewed distributions have high densities at low *t* values and low densities in the long tail of high *t* values. Scaling by *t* a positively skewed PDF flattens it, potentially enabling more accurate entropy and information estimates for fewer bins. Thus, when working from data generated via a process with substantial positive skewness, the logarithmic PDF might be preferred and should be explored.

Do ISI distributions have substantial positive skewness? The time between spikes has no maximum but a strict minimum; it is by definition positive and by biophysics greater than some minimum refractory period. In the majority of neuronal firing patterns examined, longer ISIs occur rarely and shorter ISIs occur frequently, suggesting ISI distributions with substantial positive skewness. Every ISI distribution fit and model we are aware of is positively skewed, except for the occasional misuse of the Gaussian distribution (A Gaussian is a poor model since it must allow for negative ISIs, particularly when the mean is less than 3 to 5 standard deviations, which it typically is. Apparently normal ISI data are usually better fit to gamma or log-normal distributions). Because ISI distributions happen to possess substantial positive skewness, the logarithmically binned PDFs may provide less biased and less varied entropy and information estimates for fewer bins and less data than the traditionally used linearly binned PDFs.

However, just because a distribution has positive skewness, does not mean that the logarithmic PDF is an optimal way to generate density uniformity. Indeed, it is not. Better transformations always exist, and less elegantly, bins can be arbitrarily resized to enforce uniformity.

However, one cannot know the precise nature of the distribution from sampled data, and one would prefer a method generally applicable to all potentially relevant distributions. Because we measure neuronal entropy in terms of bits per spike, *i.e.*, normalized for rate, optimal binning methodology would provide entropy estimates that vary as a function of ISI distribution coefficient of variation (CV), but not as a function of rate. Thus for example, a spike train with Poisson statistics would have the same entropy per spike, regardless of the rate. The entropy per unit time can then be calculated as simply the entropy per spike, multiplied by the spike rate. The logarithmic binning of time is the only methodology that allows for this conversion without the need for ad hoc bin width modifications.

### 2.2. Benefits of the Logarithmic PDF for Neuronal Data

Four idealized ISI distribution functions were used to model neuronal behaviors (Table 1): power law and exponential, long used to model ISIs; gamma, able to adequately characterize ISIs from several distributions [17]; and periodic log-normal, used to model stochastic responses to rhythmic inputs. The distributions were converted to PDFs (Table 2) via taking the derivative of each with respect to *t* (*dF/dt*) or the base 10 logarithm of *t* (*dF/dτ*). Parameters were selected to yield ISI distributions with a mean of 25 ms (*i.e.*, mean frequency 40 Hz), and a CV between 0.5 and 0.9.

**Figure 1:**
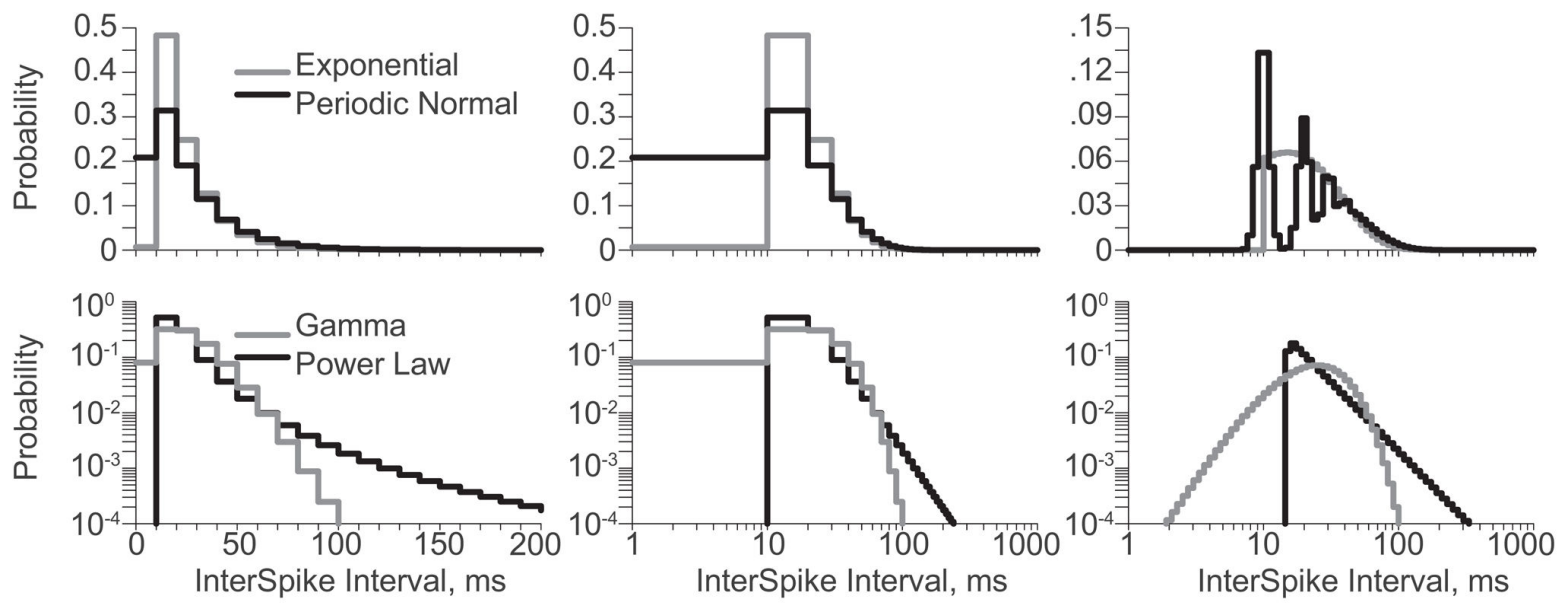
Binned PDFs for the exponential and periodic log-normal (*top*) and the gamma and power law (*bottom*) distributions. The linear PDF on a linear abscissa (*left*) and the logarithmic PDF on a logarithmic abscissa (*right*) are both straight-forward representations of the native distributions. However, the linear PDF on a logarithmic abscissa (*middle*) presents unintuitive weights that do not visually integrate to one.

The PDFs were divided evenly into 100 bins from 0.1 to 1,000 ms. The linear PDFs (Figure 1, *left*) exhibit substantial positive skewness. Problematic for information measure estimates, these discretized PDFs are dominated by a few (3–5) high probability bins (P > 0.1), a handful (5–10) of moderate probabilities bins (0.1 > P > 0.001) and overwhelming many low probability bins (P < 0.001). Also, the linear PDFs look surprisingly similar on both linear (*top*) and logarithmic (*bottom*) ordinates because poor binning has obscured the salient features of the distributions. For example, while the particular probabilities of the first three bins vary some, the shape of the exponential and periodic log-normal distributions appear unfortunately similar. The periodicity of the periodic log-normal PDF has been lost to poor binning because the bin size happens to align perfectly with the oscillation period. This problem could be overcome by increasing the number of bins, but at the expense of increasing the data needed to estimate their associated probabilities. More generally, because distribution features are rarely known a priori, appropriate bin sizes must be determined post hoc. Expanding the linear PDF by plotting it on a logarithmic abscissa (Figure 1, *middle*) does not change the discretization and thus cannot improve the numerical representation. Worse still, it distorts the visual representation (Discretizing a distribution linearly but presenting it on a logarithmic abscissa is misleading. In Figure 1, whether the linear (*left*) or the logarithmic (*right*) PDFs are integrated with respect to their own abscissas, the results is unity as the integral of any PDF must be. However, the integral of the linear PDFs with respect to logarithmic time (*middle*) is not unity).

In contrast, the logarithmically discretized PDFs (Figure 1, *right*) are much closer to flat with many (25–35) moderate probability bins (0.1 > P > 0.001). Indeed, only the power law PDF has any high probability bins (P > 0.1). The logarithmic PDFs capture the salient features of each distribution, making the four of them easily distinguishable. For example, the periodic log-normal PDF exhibits three well defined modes, and is quite distinct from the exponential PDF on the same axis. Because the salient features of the distribution are likely relevant for information transmission, the logarithmic PDFs enable information estimations that the linear distributions forbid. For example, the periodic-log normal distribution models a neuron spiking 40 times per second, but phase locked to a 100 Hz signal. Shown in the logarithmic PDF, ISIs are likely to be ~10 ms or ~20 ms, but hardly ever ~15 ms. In fact, ISIs of 15 ms are so rare that they convey ~10 bits of self-information. In contrast, from the linear PDF, ~15 ms ISIs appear extremely common and convey only ~1.5 bits of self-information!

Distributions were constructed from four parameter settings (Table 3). The first row ***(a)*** of each reports the original parameters (Figure 1). The second row ***(b)*** reports parameters for distributions with a longer mean ISI, but the same CV as ***(a)***. The third row ***(c)*** reports parameters for distributions with the same mean ISI as ***(a)***, but with a reduced CV. The fourth row ***(d)*** reports parameters for distributions with the same ISI standard deviation as ***(a)***, but the same CV as ***(c)***. Final constraints of the power law, exponential and periodic log-normal distributions were that the scale parameters of ***(b)*** and ***(c)*** equaled each other. The final constraint for the gamma distribution was matching the mean rates of ***(b)*** and ***(d)***.

**Figure 2:**
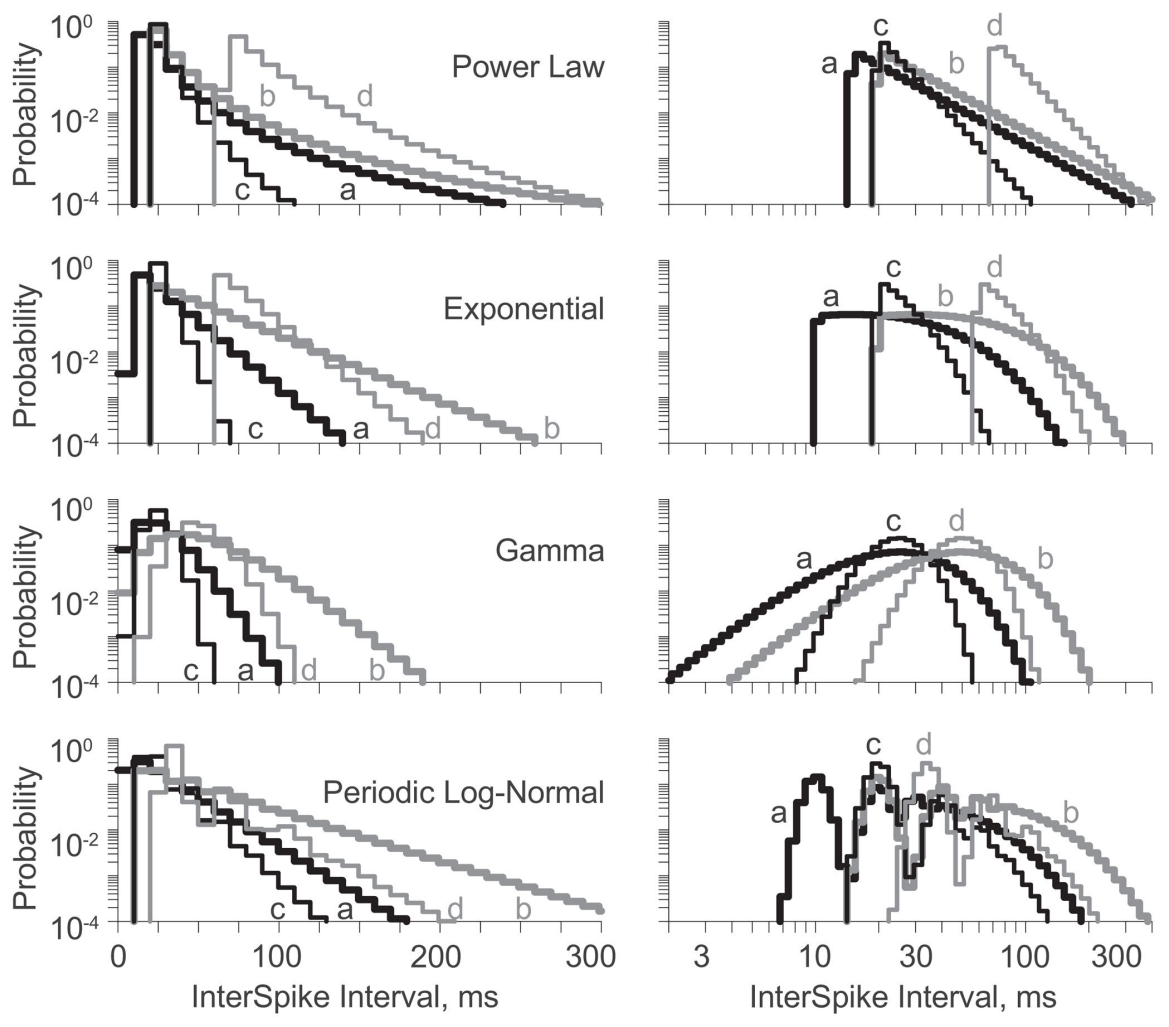
The linear (*left*) and logarithmic (*right*) binned PDFs for four parameterizations of each distribution, identified by distribution name and a letter (*a–d*) corresponding to the identifiers provided in Table 3. Note pure shifts in the logarithmic PDFs of *a:b* and *c:d*.

As before, the linear and logarithmic PDFs were divided into 100 bins (Figure 2). Note that the distributions with equivalent CVs [*i.e.*, ***(a):(b)*** and ***(c):(d)***] have logarithmic PDFs with identical shapes shifted along the abscissa. The CV quantifies the width of the logarithmic PDF exactly as the standard deviation quantifies the width of the linear PDF. If the distribution variabilities were independent of their means, the linear PDFs would shift with standard deviation as do logarithmic PDFs with CV. But, distributions with equal standard deviations (*i.e.*, ***(a):(d)***) do not have identically shaped linear PDFs.

Entropies were calculated from the discretized PDFs (Table 3, *right*). Entropies found from the logarithmic PDFs were equivalent for distributions with equal CVs [*i.e.*, ***(a):(b)*** and ***(c):(d)***]. Thus, the entropy per spike did not depend on the spike rate, so long as the distribution shape was constant. In contrast, entropies found from the linear PDFs were not necessarily equivalent for distributions with equal standard deviations [*i.e.*, ***(a):(d)***], although they were similar for the exponential and gamma distributions. The salient features of the exponential distribution are maintained through parameter modifications from ***(a)*** to ***(d)***. Indeed, the linear PDFs are nearly shifted versions of one another. However, the salient features of the gamma distribution are not maintained through the parameter modification from ***(a)*** to ***(d)***, and the near equivalence of their entropies is coincidental. Infinitely many different discretized PDF pairs happen to share similar entropies, and the linear PDFs of gamma distributions ***(a)*** and ***(d)*** represent one such pair.

Why do the standard deviations not govern the entropies of the linear PDFs as well as the CVs govern the entropies of the logarithmic PDFs? Because for a given standard deviation, the shape of the PDF is inherently dependent upon the distribution mean. For other distributions with variabilities independent of their mean, e.g., Gaussian, maintaining a constant standard deviation could be used to achieve shifts in the linear PDF mean without changing its shape or entropy. However, that class of distributions is not appropriate for neuronal ISI modeling because whenever standard deviations are independent of the mean, negative ISIs must arise for short enough ISI means. Since ISIs are by definition strictly positive, their distributions cannot have independent mean and standard deviation.

### 2.3. Bins Needed to Calculate Neuronal Information

Thus far we have used 100 bins for linear and logarithmic PDF discretization, without justification. To explore the relationship between bin count and information measures, the distributions (Table 3) were discretized over the domain *t* = 0.1 to 1,000 into from 10 to 10,000 bins. Entropy was calculated for each original distribution parameterization ***(a)***. Each other parameterization ***(b–d)*** was then individually combined with the original ***(a)*** at equal weights. Thus, we model some input to a neuron determining whether the next ISI should be drawn from distribution ***(a)*** or from the other distribution ***(b–d)***, and that both values of that input occur with probability 0.5. The mutual information that each ISI contains regarding its parent distribution identity, and thus the input parameter, was calculated for both linear and logarithmic PDFs. To maintain measure monotonicity as a function of bin number, the starting bin positions were dithered and the information measures found from each were averaged together (To maintain monotonicity of all measures, entropy and information calculations were repeated 10 times for 10 equally spaced bin shifts, and averaged together to yield the reported values. For example, for the linear PDFs with 100 bins, all bins were 9.999 ms wide but the first bin began at ~ 0.1,−0.9, −1.9, −2.9, −3.9, −4.9, −5.9, −6.9, −7.9 and −8.9 for the respective shifts).

**Figure 3:**
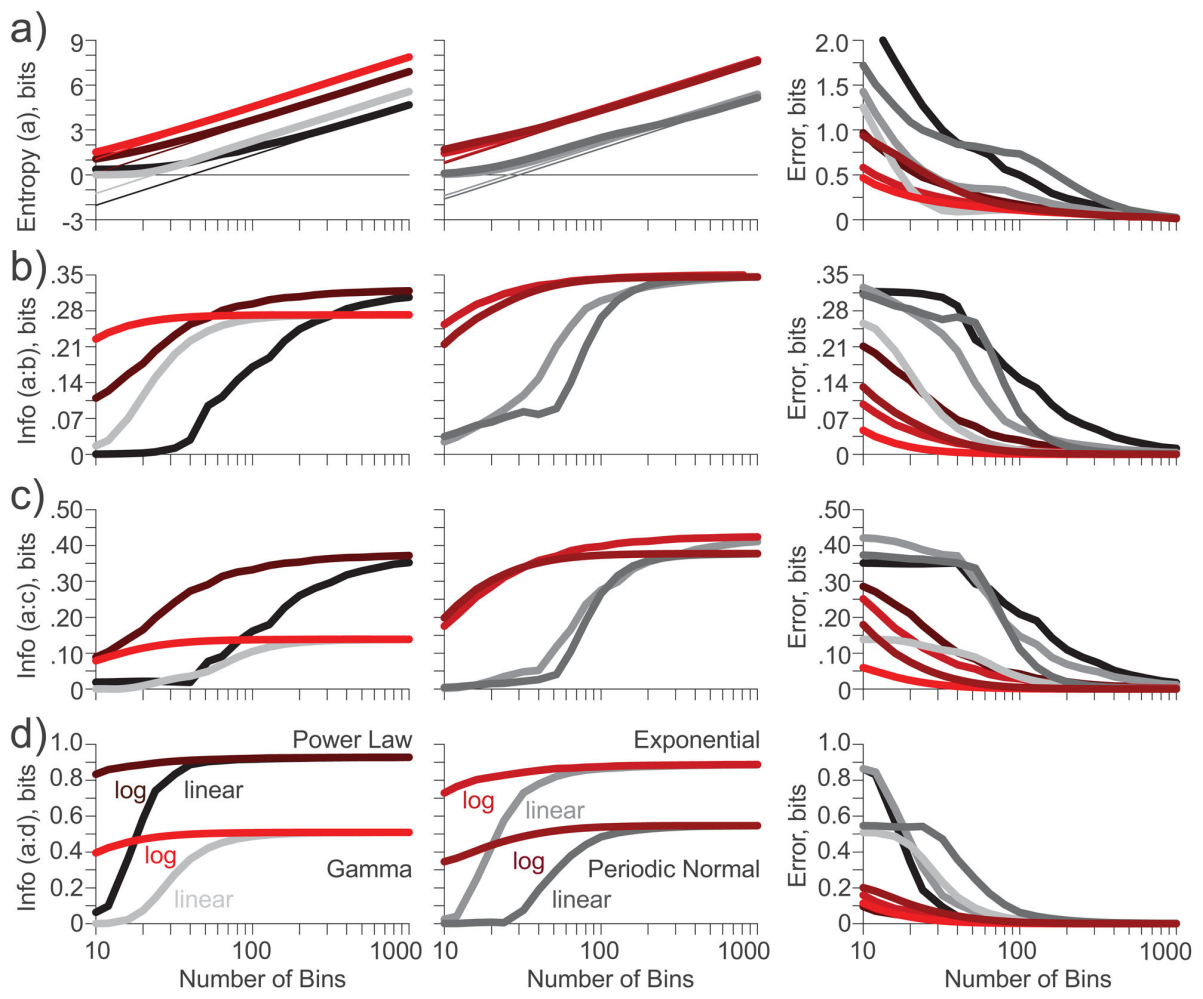
Entropy ***(a*)** and information **(*b–d*)** calculations (*left*) and binning errors (*right*) as a function of bin number for the linear (*grey-black*) and logarithmic (*red-brown*) PDFs for the four distributions and their four parameterizations provided in Table 3. Text labels identify line identities in panel **(*d*)**. (a). Entropies approach an asymptote of 1 bit per bin doubling. Entropy bin error is reported as the distance from the entropy to its asymptote; (b). Information about an input selecting for distributions ***(a)*** or ***(b)***, and the binning error; (c). Information about an input selecting for distributions ***(a)*** or ***(c)***, and the binning error; (d). Information about an input selecting for distributions ***(a)*** or ***(d)***, and the binning error.

The entropy of a discretized PDF depends on the number of bins used in the discretization (Figure 3a). For enough bins, the entropy is linearly related to the logarithm of the bin number. In particular, when a doubling in the bin number generates an entropy increase of 1.0 bits, the salient features of the PDF have been captured by the discretization. A regression line can be used to assess the sufficiency of the bin number (Figure 3a, *thin lines*). The distance from a calculated entropy to the regression line can be considered an entropy binning error, quantified in bits, and introduced by insufficient binning (Figure 3a, *right*). Entropies calculated from the logarithmic PDFs (*red-brown*) are universally closer to their asymptotes than those calculated from the linear PDFs (*grey-black*). Note that entropies from the linear and logarithmic PDFs do not asymptote to the same lines: logarithmic PDFs yield higher entropies.

Subsequent information calculations can only be as good as the underlying entropy calculations. If the bins used are not enough for the entropy estimates to asymptote to their regression line, the calculated information will be reduced relative to the true information. This reduction is neither variability nor bias, and no amount of data sampling can correct for it. If the salient features of the distribution cannot be captured by the binned PDF, all information calculations will asymptote to an incorrect value. To demonstrate this relationship, the original distributions ***(a)*** were combined with the other parameterization with equal probability to enable information calculations. We assumed that some unknown input drove the neuron to generate an ISI from distribution ***(a)*** or distribution ***(b–d)*** (Figure 3b–d, respectively), with equal probability. Thus, if the two distributions were identical, mutual information would be 0 bits; if the two distributions were exclusive, information would be 1 bit. Given that all distributions had some degree of overlap, all informations should be between 0 and 1 bit.

Unlike the entropies, the informations asymptote to a constant value, independent of bin number. Furthermore and thankfully, information calculated from the linear and logarithmic PDFs approach the same value. Thus, we are assured that regardless of our binning technique, we can measure all of the information and report the same values, so long as we have enough bins and enough data to fill them with appropriate probabilities. However, the logarithmic PDFs yield information values approaching the true value with far fewer bins than the linear PDFs in every case. With only 10 bins, most of the logarithmic PDF information values are nearing their asymptote, while the linear PDF information values are essentially zero. Quantifying the distance of an information measure to its asymptote, we calculate the information binning error, also quantified in bits, and introduced by insufficient binning (Figures 3b–d, *right*). Note that the information binning error nears zero for the logarithmic PDFs by 100 bins. Around ~700 bins would be needed to achieve equally correct information from the linear PDFs.

### 2.4. Information *versus* Inter-Spike Interval Distribution Distance

The parameters of the four distributions were modified under two different constraints, to assess the relationship between distribution parameters and information measures from the linear and logarithmic PDFs. Since 100 bins was sufficient for accurate information calculations on the logarithmic PDFs in the previous section, both PDFs were discretized into 100 bins. Differences in the information calculated from the two PDFs is the error corrected by the move from linear to logarithmic binning.

**Figure 4:**
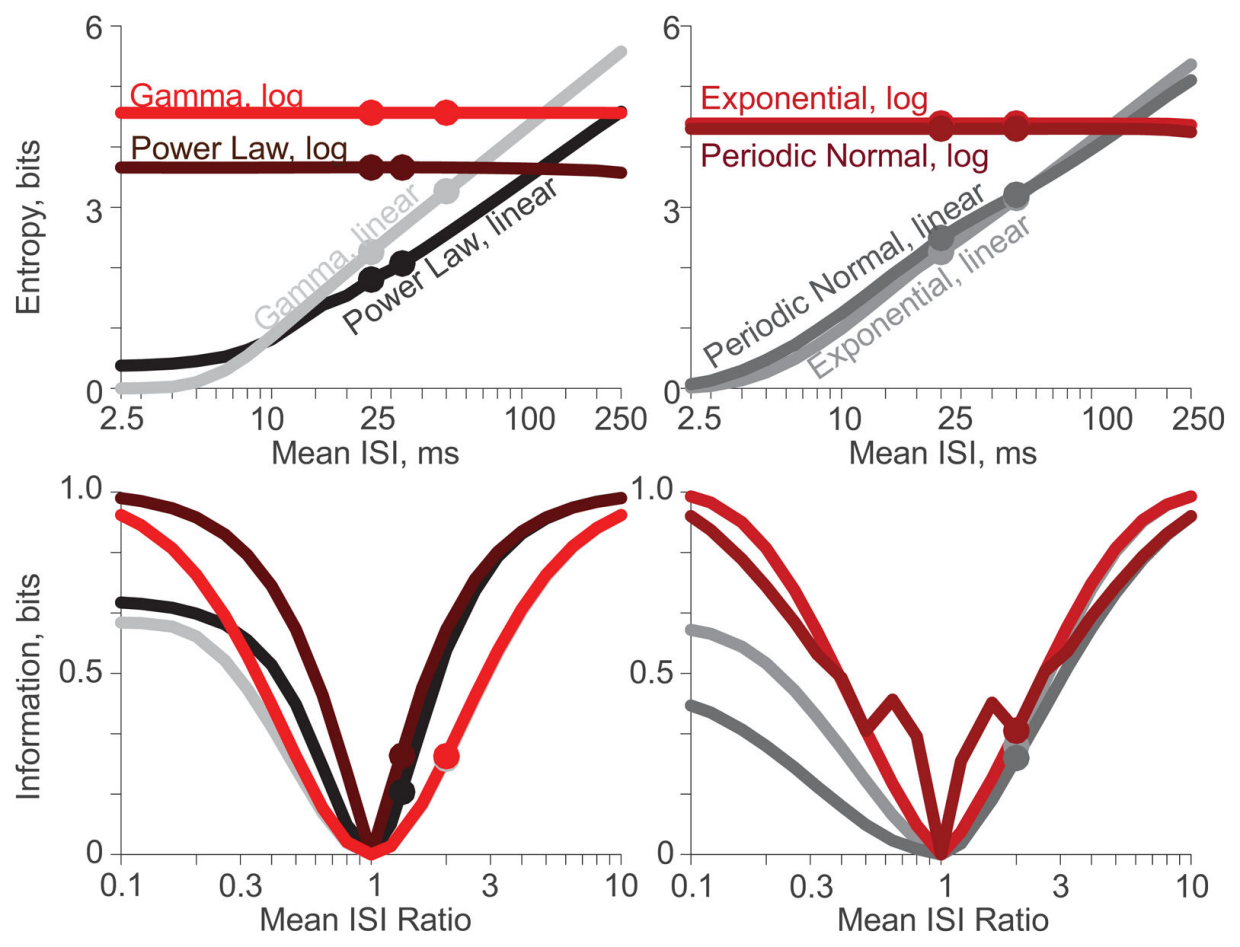
Entropy (*top*) and information (*bottom*) calculations as a function of ISI mean for the linear (*grey-black*) and logarithmic (*red-brown*) PDFs discretized into 100 bins each. Circle markers denote the parameterizations from Table 3 (a) and (b). (a). Entropies of the logarithmic calculations are independent of mean ISI; (b). Information about an input selecting for the 25 ms mean parameterization ***(a)*** or another distribution with 1/10^th^ to 10 times the mean ISI. Linear PDFs yield inferior information for short ISIs.

**Figure 5:**
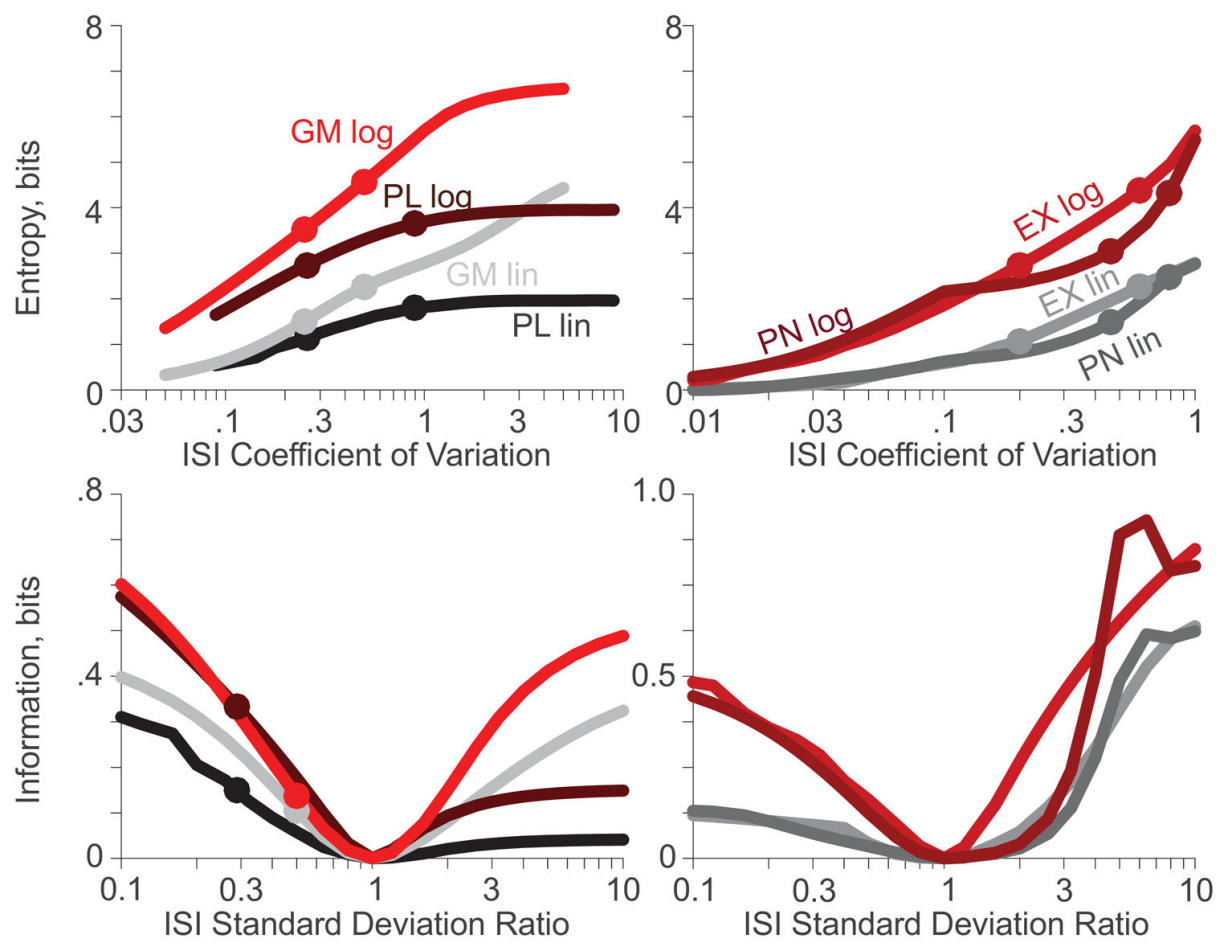
Entropy (*top*) and information (*bottom*) calculations as a function of ISI CV for the the linear (*grey-black*) and logarithmic (*red-brown*) PDFs discretized into 100 bins each. Markers denote the parameterizations from Table 3 (a) and (c). (a). Entropies from the logarithmic PDFs are always larger than from the corresponding linear PDFs; (b). More information can be recovered from the logarithmic PDFs than from the linear PDFs for nearly all distributions at all ratios of ISI standard deviation (a.k.a., all ratios of CV).

To begin we assessed how maintaining a constant CV while spanning the ISI mean across two orders of magnitude affected entropy and information measures. To generate experimental parameterizations, the CVs of all four distributions were held at their original values ***(a)***, while the means were varied from 2.5 ms to 250 ms; recall that the original distributions had means of 25 ms. To achieve this range of means, the scale parameters varied as such: power law *t_0_* ranged from 1.5 to 150 ms; exponential *t_1_* ranged from 1 to 100 ms where *λ* equaled 2/3*t_1_*; gamma *θ* ranged from 5/8 to 500/8 ms; and periodic log-normal *μ* ranged from 1 to 100 ms. Entropy was calculated from both the linear and logarithmic PDFs for all values (Figure 4, *top*). Sixteen circle markers identify parameterizations ***(a)*** and ***(b)*** from Table 3 for both discretizations of all four distributions (Upon close inspection, the markers in figure 4 match the values reported in Table 3, save for the entropies from the linear PDFs of the power law distribution; Table 3 was constructed with bins starting at 0.1 ms, whereas Figures 3–5 were averaged from dithered bins. The alignment of *t_0_* with the bin edges has a profound effect on power law entropy). While entropies of the linear PDFs increased approximately linearly with the logarithm of the ISI mean, entropies of the logarithmic PDFs were independent of mean, suggesting time-warp invariance.

The experimental parameterizations were paired with the corresponding original parameterization ***(a)*** with equal probability to assess the information calculable as a function of the ratio of experimental mean to original mean (Figure 4, *bottom*). Eight circles identify information provided by an input selecting from parameterizations ***(a)*** and ***(b)***, for both discretizations of all four distributions. When the experimental parameterization had a mean of 25 ms, it matched the original parameterization, the mean ISI ratio was one, and the information was zero. The information from each logarithmic PDF is reassuringly symmetric about this point. In contrast, information from each linear PDF falls off for small ISI ratios, where the salient features of the experimental parameterizations are not captured by the relatively large bins. Furthermore, the peaks in the information of the logarithmic PDFs of the periodic log-normal distributions (Figure 4, *bottom*-*right*) occur when the high probability modes of one parameterization coincide with the low probability troughs of the other parameterization. The linear PDFs miss the fine detail required to generate these information peaks.

Note that for ISIs larger than about 150 ms, entropy estimates from the linear PDFs exceed those from the logarithmic PDFs (Figure 3a). This peculiarity follows from poor bin alignment: recall that to keep comparisons as fair as possible, 100 bins were split from 0.1 to 1000 ms in all cases. Under experimental conditions, the bin range would align with the data, and the logarithmic entropies would remain larger than the linear entropies. As a concrete example, consider the power law distribution with a mean of 150 ms. The first 73 logarithmic bins have *zero* probability, while only the first 2 linear bins have zero probability. This massive misalignment could be fixed by shifting to the right, either the minimum ISI bin or the entire bin range. Shifting the minimum ISI bin two decades (*i.e.*, from 0.1 to 10 ms) exactly doubles the logarithmic entropy without changing the linear entropy. On the other hand, shifting the entire range two decades (*i.e.*, from 0.1→1,000 ms to 10→100,000 ms) shifts the curves in figure 3a two decades to the right. Thus, the logarithmic entropy is unaffected, but the linear entropy is driven to nearly zero. With either shift, the logarithmic entropy far exceeds the linear entropy. Examples aside, the distributions yield equal information (Figure 3b). The salient features of the large mean ISI cases can be captured by both linear and logarithmic binning, for a reasonable bin range.

Next we assessed how maintaining a constant mean while spanning the CV across two orders of magnitude affected entropy and information measures. To generate experimental parameterizations, the mean ISIs of all distributions were held at their original value (25 ms), while the CVs were varied. The power law distribution (*PL*) had CVs from 0.04√5 to 4√5 by ranging *α* from 2 + 3√14 to 2 + 9√5/20. The gamma distribution (*GM*) had CVs from 0.05 to 5 by ranging *ξ* from 400 to 1/25, with *θ* equal to 25/*ξ*. The Poisson nature of the exponential and periodic log-normal distributions restricted their CVs to below 1. Thus, to span two orders of magnitude, these distributions were set to scale their CVs from 0.01 to 1. For the exponential distribution (*EX*), scaling was done by ranging *λ* from 4 to 1/25, with *t_1_* set equal to 25−1/*λ*. The periodic log-normal distribution (*PN*) has a minimum CV when there is only one mode, in which case the CV is defined as *σ* −1. Thus, parameterizations with *σ* ≡ 1.1 cannot yield CVs less than 0.1. Therefore, two different scalings were required to range the periodic log-normal distribution from 0.01 to 1. For CVs from 0.01 to 0.1, *ρ* was set to 1 and *σ* ranged from 101/100 to 11/10. For CVs from 0.1 to 1, *σ* was set to 11/10 and *ρ* ranged from 1 to 1/20. Entropies were found for all experimental CV parameterizations (Figure 5), as was done previously for the experimental means.

For each distribution, a default parameterization was identified as that with its CV at the geometric mean of the CV range. For the power law and gamma distributions, the default parameterizations were the original parameterizations ***(a)***. For the exponential and periodic log-normal distributions, the default parameterizations were those yielding CVs of 0.1. Distributions at each CV were combined with the default distribution with equal probability to assess the information calculable as a function of the ratio of CVs (Figure 5, *bottom*). Note that since all parameterizations had the same mean ISI, the ratio of CVs is equivalent to the ratio of standard deviations. Four circle markers denote the information calculated in Figure 3 for the power law and gamma distributions (Figure 5, *bottom-left*). Corresponding markers are not shown for the exponential and periodic log-normal distributions because their default parameterizations were not explored in Figure 3.

When the experimental parameterization had the same CV as the default parameterization, the standard deviation ratio was one and the information was zero. However, the information was not symmetric about this point as a function of CV ratio. While the details of each curve depended critically upon the distribution, information calculated from the logarithmic PDFs was substantially improved over information calculated from the linear PDFs. If the CVs were at all different, and thus capable of providing information, the logarithmic PDFs yielded superior information measures.

## 3. Conclusions

Information theory has long been recognized for its utility to neural analysis [1], and for the framework it provides to quantitatively address concepts of brain processing. Beyond mammalian sensory and motor processing [3–7], it has helped answer how basic neurons process information (e.g., via application to model systems such as fly vision [18] and the electric sense of electric fish [19]), how large neuronal networks are connected [20], and how brain circuits weigh the potential rewards of a behavior against the effort it requires [21]. And yet, despite theoretical advances [14,15,22], and the recent dissemination of practical recipes [23–25] and computational tools [26–29], information theory remains a niche subfield within neuroscience. The future spread of information theory requires continued development and straightforward comparisons of the benefits of different approaches [30].

While a variety of sophisticated entropy and information estimation techniques exist, this manuscript is relevant to so-called *direct* techniques, in which the spike times are discretized and binned. Previous work has explored the benefits of logarithmically binned ISI data for identifying neuronal bursting activity [12] and estimating entropy with minimal bias and variability [11]. Neuronal ISI distributions are strictly positive, and include a long tail of low probability for long ISIs. These characteristics underlie distributions with positive skewness, compactly represented on a logarithmic abscissa. Thus, the logarithmic PDF is a more natural representation of neuronal ISIs than the linear PDF. However, though the concept of *natural* representations may appeal to our intuition, a particular representation is irrelevant so long as we are not misled. More importantly, if the data are to be used to estimate some underlying quantities, the preferred representation should yield the best estimates.

For a variety of distributions used to model neuronal ISIs, we contrasted entropy and information calculations made from binning the linear and logarithmic PDFs. Entropy and information approached their asymptotic values for fewer bins with the logarithmic PDFs. Note that finer bin resolution could be provided to both PDFs by restricting the ISIs to a smaller domain. However, in the experimental world, that domain is provided by the data, and the larger the ISIs, the grosser the bin resolution. In particular, bin resolution of the logarithmic PDF is less sensitive to anomalously long ISIs than the linear PDF. For example, imagine thousands of ISIs collected from a neuron ranged from 0.1 to 1000 ms, until one anomalous ISI lasted 2,000 ms, twice the previous maximum. To maintain the same number of bins, the bin width of the logarithmic PDF would have to be increased by just under 10%, whereas the bin width of the linear PDF would have to be doubled.

The number of bins used to discretize neuronal ISIs must be enough to capture the salient features of the distribution that vary across conditions; if fewer bins are used, no amount of data will be enough to yield accurate information estimates. However, more bins require more data to accurately calculate the bin probabilities; if the bin probabilities are not calculated sufficiently, information estimates will include positive bias. Thus, an experimentalist should aim to use just enough bins to capture the distribution salience, and at least enough data to estimate sufficiently the probabilities associated with those bins. In earlier work, we have shown that for a fixed amount of data, and a fixed number of bins, the logarithmic ISI distributions yield entropy estimates with less bias and less variance [11]. We now add that logarithmic ISI distributions enable better discretization for fewer bins, generating more accurate information estimates. Thus, with logarithmic ISIs: the distribution salience is expressed in fewer bins, the bin probabilities are estimated with less data, and the entropy estimates include less bias and variability. In combination these finding suggest that information estimates from logarithmically binned ISIs approach their true value for far less data than from linearly binned ISIs. Future work should explore in detail how bin size interacts with data sampling, and the relative contributions of underbinning and undersampling bias to the final information estimates.

Linear PDFs yield entropies that, beyond some minimum duration required to capture the distribution salience, increase linearly as a function of mean ISI. However, logarithmic PDFs yield entropies that are independent of mean ISI (Figure 4). In this regard, the entropy of the logarithmic PDF is time-warp invariant. Several studies have verified time-warp invariance in the neural processing of auditory systems, from variable speed song identification in the grasshopper [31,32], to variable rate speech interpretation in humans [33,34]. Linear and logarithmic PDFs yield identical information measures, so long as the salient features of the distributions are captured by the binning. However, for a fixed bin number, the logarithmic PDFs are more likely to capture salient distribution features, be they short ISIs or periodicity, and thus yield higher, better information measures. Future work will continue to explore these findings in conjunction with how the brain combines information across neurons.

## Figures and Tables

**Table 1:** Cumulative distributions (***F(t)***) of the 4 examples used in this manuscript. Abbreviations: natural logarithm *ln()*, base 10 logarithm *log()*, error function *erf()*, regular gamma function *Γ()*, and incomplete gamma function *γ()*.

		*F(t)*
Power Law (*t_0_*, *α*)	*t* ≤ *t_0_*	0
	*t > t_0_*	1 −(*t/t_0_*)^−^ *^α^*^+1^
Exponential (*t_1_, λ*)	*t* ≤ *t_1_*	0
	*t > t_1_*	1 − *e*^− *λ*(*t*− *t*_1_)^
Gamma (*θ, ξ*)	*t* ≤ 0	0
	*t >* 0	$\frac{\gamma (\xi ,t/\theta )}{\Gamma (\xi )}$
Periodic Log-Normal (*μ*, *ρ, σ*)	*t* ≤ 0	0
	*t >* 0	$\frac{\rho }{2}\sum_{k=1}^{\infty }{\left(1-\rho \right)}^{k-1}\left(1+\mathrm{erf}\left(\frac{\log(t/\mu k)}{\sqrt{2}\log(\sigma )}\right)\right)$

**Table 2:** The example linear (***dF/dt***) and logarithmic (***dF/dτ***) PDFs.

		*dF/dt*	*dF/dτ*
Power Law	*t > t_0_*	$(\alpha -1)t^{-\alpha }/t_{0}^{-\alpha +1}$	*ln*(10) (*α* − 1)(*t/ t_0_*)^−^ *^α^*^+1^
Exponential	*t > t_1_*	*λe*^− *λ*(*t*− *t*_1_)^	*ln*(10) *λte*^− *λ*(*t*− *t*_1_)^
Gamma	*t >* 0	$\frac{t^{\xi -1}e^{-t/\theta }}{\theta^{\xi }\Gamma (\xi )}$	$ln(10)\;\;\;\frac{t^{\xi }e^{-t/\theta }}{\theta^{\xi }\Gamma (\xi )}$
Periodic Log- Normal	*t >* 0	$\frac{\rho /(\mathrm{tln}(10))}{\sqrt{2\pi }\log(\sigma )}\sum_{k=1}^{\infty }{\left(1-\rho \right)}^{k-1}e^{-{\left(\frac{\log(t/\mu k)}{\sqrt{2}\log(\sigma )}\right)}^{2}}$	$\frac{\rho }{\sqrt{2\pi }\log(\sigma )}\sum_{k=1}^{\infty }{\left(1-\rho \right)}^{k-1}e^{-{\left(\frac{\log(t/\mu k)}{\sqrt{2}\log(\sigma )}\right)}^{2}}$

**Table 3:** Parameter values for four instantiations of each distribution, with distribution mean (1,000/rate), standard deviation (STD) and CV. Entropy was calculated for all distributions from the linear and logarithmic probability densities divided into 100 bins each.

		*Scale Parameter*	*Shape Parameter*	*Average Rate, Hz*	*ISI STD Dev., ms*	*ISI CV*	*Linear Entropy, bits*	*Logarithmic Entropy, bits*
Power Law	*(a)*	*t_0_ =* 15	α = 7/2	40	10√5	2/√5	1.87	3.68
	*(b)*	20	7/2	30	200/√15	2/√5	1.79	3.67
	*(c)*	20	6	40	25/√15	1/√15	0.71	2.78
	*(d)*	40√3	6	20/√3	10√5	1/√15	2.38	2.75
Exponential	*(a)*	*t_1_* = 10	*λ* = 1/15	40	15	3/5	2.09	4.38
	*(b)*	20	1/30	20	30	3/5	3.04	4.38
	*(c)*	20	1/ 5	40	5	1/5	0.67	2.76
	*(d)*	60	1/15	40/3	15	1/5	2.07	2.77
Gamma	*(a)*	*θ* = 25 / 4	*ξ* = 4	40	25/2	1/2	2.32	4.56
	*(b)*	25 / 2	4	20	25/1	1/2	3.27	4.56
	*(c)*	25 /16	16	40	25/4	1/4	1.51	3.52
	*(d)*	25 / 8	16	20	25/2	1/4	2.39	3.52
Periodic Log- Normal- *σ = 1.1*	*(a)*	*μ* = 10	*ρ* = 2/5	40	19.69	0.788	2.68	4.29
	*(b)*	20	2/5	20	39.38	0.788	3.44	4.29
	*(c)*	20	4/5	40	11.54	0.461	1.91	3.04
	*(d)*	34.14	4/5	23.43	19.69	0.461	1.78	3.04
